# Monitoring of Antibiotic Resistance Patterns Within Al-Karak Governmental Hospital, Jordan, in 2022

**DOI:** 10.3390/antibiotics13121172

**Published:** 2024-12-04

**Authors:** Amin A. Aqel, Tala M. Al-Matarneh, Tayf K. Al-Tarawneh, Tahrir Alnawayseh, Mohammed Alsbou, Yasser Gaber

**Affiliations:** 1 Department of Microbiology and Immunology, Faculty of Medicine, Mutah University, Al-Karak 61710, Jordan; aminaq@mutah.edu.jo; 2 Department of Pharmacology and Toxicology, Faculty of Pharmacy, Cairo University, Cairo 12613, Egypt; tala.matarneh@yahoo.com; 3 Department of Pharmaceutics and Pharmaceutical Technology, Faculty of Pharmacy, Mutah University, Al-Karak 61710, Jordan; taifkhaledtara@yahoo.com (T.K.A.-T.); yasser.gaber@pharm.bsu.edu.eg (Y.G.); 4 Infection Prevention and Control Al-Karak Governmental Hospital, Al-Karak 61710, Jordan; tahreer761@yahoo.com; 5 Department of Pathological Sciences, College of Medicine, Ajman University, Ajman P.O. Box 346, United Arab Emirates; 6 Department of Pharmacology, Faculty of Medicine, Mutah University, Al-Karak 61710, Jordan; 7 Department of Microbiology and Immunology, Faculty of Pharmacy, Beni-Suef University, Beni-Suef 62511, Egypt

**Keywords:** WHO AWaRe, bacterial isolates, antibiotic resistance patterns, Jordan, Al-Karak Government Hospital

## Abstract

**Background/Objectives**: Antimicrobial resistance is considered one of the foremost global public health challenges, and its prevalence is increasing. In Jordan, particularly in Al-Karak Governorate, there is a lack of sufficient data on antimicrobial resistance to make accurate assessments. The main aim of the current study was to evaluate antibiotic resistance trends in clinical specimens from 2022 and assess antibiotic resistance patterns. The emphasis on the WHO antibiotic classification as Access, Watch, and Reserved (AWaRe) was adopted in the current study. **Results**: Among Gram-positive bacteria, *Enterococcus faecalis* exhibited 100% susceptibility to nitrofurantoin and 96% to vancomycin, *Streptococcus viridans* exhibited 100% susceptibility to teicoplanin, while CoNS (coagulase-negative *Staphylococci*) showed moderate resistance to Trimethoprim + Sulfamethoxazole (63%) and clindamycin (47%). Among Gram-negative bacteria, *Escherichia coli* and *Klebsiella pneumoniae* displayed high susceptibility to fosfomycin (*E. coli*: 95%, *K. pneumoniae*: 80%) and amikacin (*E. coli*: 93%, *K. pneumoniae*: 81%). Resistance was notable for trimethoprim + sulfamethoxazole (*E. coli*: 47%, *K. pneumoniae*: 53%) and nitrofurantoin (*K. pneumoniae*: 30%). *Pseudomonas aeruginosa* exhibited the highest proportion of XDR strains (15%), followed by *K. pneumoniae* (11%) and *E. coli* (4%), while PDR strains were found in *P. aeruginosa* (6%), *K. pneumoniae* (3%), and *E. coli* (0.6%). XDR was observed in 4% of CoNS and 3% of *S. viridans* (α), with *Staphylococcus aureus* exhibiting both XDR and PDR at 1%. **Methods**: A cross-sectional retrospective study of bacterial species and their antimicrobial susceptibility was carried out at a hospital in Al Karak, Jordan, from January to December of 2022, the study included 1187 isolates from all locations in Al-Karak Governmental Hospital. **Conclusions**: The significant prevalence of XDR and PDR strains in key pathogens, particularly *P. aeruginosa* and *K. pneumoniae*, underscores the need for a robust Antimicrobial Stewardship Program (ASP) and infection control measures at Al-Karak Governmental Hospital. High susceptibility in several Access group antibiotics (e.g., amikacin and nitrofurantoin) supports their prioritization in empirical therapy, while the emergence of resistance in Watch and Reserved antibiotics highlights the necessity for rational use. These findings are very important for adjusting the local strategies to lower the spread of resistant strains and improve clinical outcomes.

## 1. Introduction

The worldwide threat of antibiotic resistance is exacerbated by the advent of multidrug-resistant (MDR) bacteria, which spreads the problem faster than the creation of new medicines to treat resistant diseases [1,2]. Because of their resistance, clinically available antibiotics are ineffective against these strains, rendering antibacterial therapy ineffective in treating associated infectious episodes. Antibiotic overuse and a lack of adequate antibiotic stewardship programs are the main causes of antibiotic resistance. Antibiotic resistance kills 700,000 people worldwide each year, and if no action is taken, this number is expected to triple by 2050 [3].

The Hashemite Kingdom of Jordan, situated in the Eastern Mediterranean Region (EMR), exhibits substantial pervasiveness of self-medication with antibiotics at 40% [4]. The World Health Organization (WHO) has identified the EMR as an area with a high likelihood of antibiotic misuse. It is challenging to evaluate and effectively address the issue of the precise prevalence of antibiotic resistance in the region due to the absence of accurate surveillance mechanisms for antibiotic resistance [5].

Despite the global focus on antibiotic resistance, a significant knowledge gap regarding the specific challenges faced by certain regions remains. Most of the existing research comes from wealthy countries, leaving resource-limited settings like Jordan underrepresented. Consequently, there is a lack of comprehensive data and insights into the prevalence, trends, and drivers of antibiotic resistance in this area. To address this issue, more survey studies are needed to mitigate the rise in antibiotic resistance. Additionally, there is a lack of information on antibiotic resistance in South Jordan, with only a limited sample size available [6,7]. This scarcity underscores the urgent need for this study.

Antibiotic Stewardship Programs (ASPs) are hospital-based interventions aimed at improving antibiotic use. The CDC recommends them to control antibiotic consumption in acute care hospitals. ASPs promote the best antibiotic regimens considering factors like dosage, treatment duration, and administration mode. Jordanian hospitals have been evaluated for their implementation of ASPs, demonstrating the importance of a multi-faceted approach to reducing antibiotic resistance [8]. However, data on Jordanian practitioners’ current attitudes and behaviors toward antimicrobial stewardship initiatives is limited. Shedding light on these aspects reveals gaps in knowledge and the use of antimicrobial stewardship programs (ASPs) and offers guidance for promoting strategies such as delaying antibiotic prescriptions. This understanding will help establish a foundation for implementing these programs in Jordanian healthcare institutions [8].

The World Health Organization has advised applying (AWaRe) classification to promote antibiotic stewardship at the local, national, and international levels. Access, Watch, and Reserve are the newly created categories for common antibiotics (AWaRe). Compared to other groups, antibiotics within the “access” classification are antibiotics with a narrow spectrum of activity, often fewer adverse effects, a lesser risk of antimicrobial resistance developing, and a cheaper cost. They need to be widely accessible and advised for the empirical treatment of the majority of common infections. Antibiotics in the “watch” group are more frequently used in sicker patients in the hospital facility setting and have a larger potential for the selection of antimicrobial resistance, to prevent misuse, their usage should be closely monitored. Antibiotics in the “reserve” group are last-resort medications that need to be reserved for the treatment of serious illnesses brought on by multidrug-resistant pathogens [9].

## 2. Results

The current study is based on the “Antibiogram, Al-Karak Hospital, Ministry of Health, 2022” report. This report included susceptibility data that was extracted from the electronic national registration database known as Hakeem. It provided the susceptibility patterns among different pathogens recorded in Al-Karak Governmental Hospital in 2022. The main findings of the report are presented in the following tables. Table 1 illustrates the distribution of bacterial isolates by specimen type, revealing that urine samples accounted for the largest proportion, comprising 61% of all isolates. The remaining percentage (39%) refers to the other types of sample sources, i.e., blood, vagina, skin, tissue, and respiratory samples. These results revealed a wide variety of bacterial diseases impacting different bodily systems. Therefore, Table 1 offers a comprehensive overview of the bacterial isolates from various sources, guiding targeted diagnostic and treatment strategies while providing valuable insights into the distribution of infections across different specimen types.

Table 2 shows Gram-positive bacteria reported in 2022 at Al-Karak Governmental Hospital. Those were mainly *S. viridians* (α), *E. faecalis*, and CoNS (Coagulase-negative *Staphylococci*) isolated from urine specimens. A total of 69 urine isolates were reported in the “Antibiogram, Al-Karak Hospital, Ministry of Health, 2022” report.

The table also shows the categories of antibiotics according to the WHO AWaRe scheme in collaboration with the Jordanian Health Authority. The “Access” antibiotics class in the case of urine isolates are many compared to the Watch and Reserve groups, suggesting that these treatments are suitable and accessible for empirical use. In some cases, however, antibiotics such as cefepime, levofloxacin, and others need to be used with caution (“Watch”). Linezolid, fosfomycin, and other drugs may only be used sparingly, according to the “Reserved” categorization. The original report released by the Ministry of Health: “Antibiogram, Al-Karak Hospital, Ministry of Health, 2022” has adopted the following conventions in reporting, S1, and S2 refer to expected susceptibilities, and R, refers to expected resistance. Table 2 shows Gram-positive isolates that are susceptible (S1 and S2) to vancomycin, all isolates demonstrated susceptibility to linezolid, CoNS and *S. viridians* (α) demonstrate 100% susceptibility to teicoplanin. The susceptibility rates of ciprofloxacin and levofloxacin range from moderate to high. The efficacy of erythromycin varies depending on the isolate. Differences in susceptibility are observed in gentamicin, clindamycin, and trimethoprim + sulfamethoxazole, with *E. faecalis* demonstrating resistance to these antibiotics, specifically, *E. faecalis* exhibits a 100% susceptibility rate to nitrofurantoin and Penicillin G.

The Gram-negative bacteria isolated from urine samples according to the “Antibiogram, Al-Karak Hospital, Ministry of Health, 2022” report are shown in Table 3. A total of 655 urine isolates were included in this cross-sectional study. A distinctive susceptibility pattern emerged when assessing antibiotic susceptibility percentages for these isolates. Notably, in both *E. coli* and *K. pneumoniae*, high susceptibility was found with fosfomycin, along with other effective antibiotics such as amikacin (*E. coli*: 93%, *K. pneumoniae*: 81%), piperacillin + tazobactam (*E. coli*: 92%, *K. pneumoniae*: 81%), and gentamicin (*E. coli*: 89%, *K. pneumoniae*: 88%). *E. coli* displayed additional high susceptibility to Nitrofurantoin (86%) and Ceftolozane + Tazobactam (94%). All isolates demonstrated susceptibility to the carbapenem group (imipenem, meropenem, ertapenem) and colistin. Moreover, Tigecycline exhibited notable efficacy against most *E. coli* isolates. Gram-negative bacteria showed varying percentages of susceptibility to the following antibiotic classes: amoxicillin + clavulanic (*E. coli*: 55%, *K. pneumoniae*: 63%), trimethoprim + sulfamethoxazole (*E. coli*: 47%, *K. pneumoniae*: 53%), nitrofurantoin (*E. coli*: 86%, *K. pneumoniae*: 30%), cephalosporin group (31–74%), and fluoroquinolone group (47–69%).

This study highlights the diverse and intricate patterns of antibiotic susceptibility seen in the Gram-negative bacterial isolates, offering significant perspectives for formulating efficacious therapeutic approaches at Al-Karak Governmental Hospital.

Table 4 summarizes the classification of bacterial isolates as extensively drug-resistant (XDR) and pan-drug-resistant (PDR). The term “XDR” denotes a non-susceptibility to at least one agent in all but two or fewer antimicrobial classes (i.e., bacterial isolates stay susceptible to only one or two classes), and PDR was described as non-susceptibility to any drug across all antimicrobial classes [10].

A significant degree of resistance in these pathogens is shown by the existence of XDR strains in *Pseudomonas aeruginosa* (8 out of 53, 15%) and *Klebsiella pneumoniae* (7 out of 62, 11%). A significant proportion of XDR strains (28 out of 722, 4%), as seen in *Escherichia coli*, highlight the need to keep an eye out for resistance patterns. While *Streptococcus viridians* (α) and CoNS show limited resistance, *S. aureus* has a comparatively lower incidence of XDR strains (1 out of 76, 1%) (Table 4).

Table 5 highlights the comparative antibiotic susceptibility of Gram-positive isolates, including *E. faecalis*, *S. viridans* (α), and CoNS, between the current study and previous research. Notably, *E. faecalis* displayed lower susceptibility to erythromycin (9% vs. 62.5%) and vancomycin (96% vs. 100%) but showed higher susceptibility to ciprofloxacin (73% vs. 57.9%) and levofloxacin (77% vs. 57.9%). Similarly, *S. viridans* (α) demonstrated reduced susceptibility to erythromycin (40% vs. 61%) and clindamycin (44% vs. 89%) but higher susceptibility to levofloxacin (82% vs. 64.3%). CoNS isolates showed lower susceptibility to oxacillin (14% vs. 24.2%) but higher susceptibility to erythromycin (28% vs. 8.3%). Full details of the comparison are available in the Supplementary Information.

Table 6 shows the comparison of selected cases of antibiotic susceptibility of *E. coli* and *K. pneumoniae* isolates between the current study and previous research. *E. coli* demonstrated lower susceptibility to cefuroxime (43% vs. 60.5%), ciprofloxacin (47% vs. 75.8%), and piperacillin + tazobactam (92% vs. 95.7%), while showing higher susceptibility to nitrofurantoin (86% vs. 75%) and tigecycline (100% vs. 60%). Similarly, *K. pneumoniae* exhibited lower susceptibility to piperacillin + tazobactam (81% vs. 100%), cefuroxime (31% vs. 50%), and ciprofloxacin (48% vs. 97.3%), but higher susceptibility to nitrofurantoin (30% vs. 20.9%). The full version of this table is found in the Supplementary Information.

## 3. Discussion

The current study is a cross-sectional study that evaluated the antimicrobial susceptibility patterns recorded at Al-Karak Governmental Hospital based on the officially published Antibiogram Report 2022 issued by the Ministry of Health, Jordan, in mid-May 2023. The current study is important in addressing the antimicrobial resistance problem, providing policymakers in the south of Jordan with insights about the circulating resistant bacterial strains, and assisting in implementing effective protocols in antibiotic stewardship strategies.

The current study found that isolates from blood origin are few compared to urine samples. This might be explained by the fact that hospitals often rely on broad-spectrum or highly effective antibiotics in case of blood sepsis since these are emergencies and ignore susceptibility testing. There is usually no opportunity to wait for culture results or antimicrobial susceptibility testing before starting the treatment. However, we advise isolating the pathogen and performing suitable antimicrobial susceptibility testing for an effective antimicrobial stewardship strategy. CoNS were the predominant bacterial isolate in bloodstream infections (Table S1, Supplementary Information), aligning with findings from studies conducted in Egypt, Zimbabwe, and Gabon, where CoNS were also identified as the major isolates in such infections [17,18]. In contrast to our findings, research in Northern Jordan identified *K. oxytoca* as the primary pathogen in bloodstream infections [19]. The high percentage of CoNS may be attributed to several factors, including the increased nosocomial infection rate in our hospital [20] and inadequate hygienic care. According to a recent report, bacterial infections acquired in hospitals are most common in developing nations [21].

Urinary tract pathogens are common problematic pathogens at Al-Karak Governmental Hospital. The susceptibility results for Gram-positive pathogens isolated from urine samples showed that 80% of *E. faecalis* were sensitive to antibiotics, offering a broad range of options for empirical therapy. High sensitivity was noted for three antibiotics from the access group, vancomycin from the watch group, and linezolid from the reserved group. For *S. viridans* (α), more than 80% of isolates were sensitive to two antibiotics from the access group, four from the watch group, and linezolid from the reserved group. Similarly, the susceptibility results for CoNS indicated that over 80% of isolates were sensitive to two antibiotics in the access group, four in the watch group, and linezolid in the reserved group. According to our results, *K. pneumoniae and E. coli* were identified as the primary bacterial agents in urine specimen isolates, indicating a significant prevalence of urinary tract infections. This finding aligns with a study conducted at Prince Hashim Hospital in Zarqa, Jordan, which also reported *K. pneumoniae and E. coli as major bacterial agents* [22]. In *E. coli*, more than 80% sensitivity was observed for three antibiotics in the access group, four in the watch group, and five in the reserved group. For *K. pneumoniae*, over 80% sensitivity was reported for two antibiotics in the access group, four in the watch group, and three in the reserved group. These findings can help guide the selection of empirical therapy for UTI in Al-Karak Governmental Hospital, keeping in mind that physicians should begin with the access group, then the watch group, and finally the reserve group, considering the patient’s other factors.

In this study, involving 53 clinical isolates of *Pseudomonas aeruginosa* from patients at Al-Karak Hospital in Jordan, we identified 15% (*n* = 8) as XDR and 6% (*n* = 3) as PDR. Notably, our findings indicate a lower prevalence of XDR *P. aeruginosa* compared to those reported at Jordan University Hospital [23]. Additionally, we observed two PDR isolates, which aligns with other studies that reported frequencies of 3.8% (*n* = 2) in Spain, Greece, and Italy [21], and 2.4% (*n* = 5) in Qatar [24]. In contrast to findings from other studies, no PDR *Pseudomonas aeruginosa* isolates were reported in Iran [25]. In this study, 1% (*n* = 1) of *S. aureus* isolates were classified as XDR and another 1% (*n* = 1) as PDR. These findings are lower than those reported in early 2022 in Jordan [26] and Egypt [27]. In our study, 4% (*n* = 28) of *E. coli* isolates were classified as XDR, while only 1% (*n* = 4) were classified as PDR. These findings are lower than those reported in Iraq [26]. In this study, 4% (*n* = 2) of *Staphylococcus* CoNS isolates were classified as XDR, with no isolates identified as PDR. These findings are lower than those reported in Northern Egypt [27] and Nigeria [28]. Importantly, XDR strains account for 4% of all isolates, while PDR strains constitute 0.8%. This emphasizes the need for ongoing surveillance and strategic interventions to address the rise in highly resistant bacterial strains.

The comparisons of our susceptibility percentages to studies conducted in Jordanian settings showed lower resistance in Al-Karak Governmental Hospital among Gram-positive isolates towards ciprofloxacin, levofloxacin, and erythromycin in some cases. This indicates a need for further monitoring of these antibiotics in the hospital and potentially reducing their usage to prevent resistance escalation. Although the comparison was made against studies conducted in Jordan, reflecting the specific conditions of those studies, the findings offer valuable insights. For instance, antibiotics such as clindamycin and oxacillin require special attention due to their lower susceptibility, signaling the importance of focused efforts to ensure their continued efficacy in treating Gram-positive infections. For Gram-negative bacteria, the comparison revealed reduced susceptibility to antibiotics like ciprofloxacin and cefuroxime, especially in treating *E. coli* and *K. pneumoniae*. These findings suggest the need for enhanced monitoring and more cautious use of these antibiotics in clinical practice. However, the higher susceptibility observed for tigecycline, and nitrofurantoin provides reassurance regarding their effectiveness, making them viable options for resistant Gram-negative infections. Such targeted monitoring and careful antibiotic stewardship at Al-Karak Governmental Hospital can help address emerging resistance issues while ensuring effective patient care.

It is noteworthy to mention the spread of misuse and overuse of antibiotics in Middle Eastern countries and in Jordan specifically that results in increased risk of antibiotic resistance. Antibiotics are often dispensed without a prescription due to various factors, including pressure from patients and pharmacy owners, patients’ socioeconomic status, and profit considerations, as many individuals cannot afford physician fees [29]. Previous studies conducted in Jordan to assess the prevalence of antibiotic self-medication among Jordanians yielded similar findings. The most often used antibiotic was amoxicillin; however, only 37.6% of patients followed the recommended dosage. In addition, 40.7% of patients in Jordan have taken antibiotics without a prescription and 19.7% of patients have asked a different doctor or physician to acquire antibiotics if their first doctor has not prescribed any antibiotic [30]. In Jordan, current regulations indicate that antimicrobials can only be administered by prescription, and only physicians and dentists are authorized to prescribe antibiotics [31], this is not effectively managed, and antibiotics can still be provided without a prescription at the request of the patient, increasing the problem of antimicrobial resistance [30,31], this problem may be compounded by antibiotic abuse and overuse as a result of incorrect prescribing by clinicians who lack adequate supportive clinical information [32,33].

The current study underscores the critical importance of implementing effective antimicrobial stewardship programs to prevent the spread of drug-resistant strains across various organisms. Remarkably, the Jordanian Ministry of Health (MoH) has set procedures for the implementation of the Surgical Antibiotic Prophylaxis Program. In addition to optimizing the use of antibiotics to limit the evolution of antimicrobial resistance, this program seeks to lower the incidence of surgical site infections (SSIs), one of the most frequent postoperative consequences [34].

## 4. Materials and Methods

The data presented in the current study was extracted from the Al-Karak Governmental Hospital 2022 annual report authorized by the Ministry of Health, Jordan. The data were collected based on the MOH/REC/2023/75 agreement between Mutah University and the Jordanian Ministry of Health. The annual “Antibiogram, Al-Karak Hospital, Ministry of Health, 2022” report included a retrospective analysis of bacterial species and their sensitivity to antimicrobials during 2022. It included 1187 isolates with accessible in vitro susceptibility test results. The report depended mainly on the Hakeem database, the National Jordanian Health Registry Database [35]. The following considerations were followed to generate this annual report: susceptibility data were collected according to each device’s (e.g., Vitek 2 System, bioMérieux, Marcy L’Etoile, France) output (breakpoints) and the resources available in the lab, and the microbiologist verified the susceptibility lab test results. Then, the microbiologists summarized the results using the codes R for resisting, I for intermediate, and S for sensitive and entered them into the patient’s record in the Hakeem database. Only (S) results were considered in this study to determine the susceptibility percentages. The previous published protocols regarding the calculation of aggregations and sensitivity percentages was followed; specifically, one-year (365 days) window was considered to calculate the sensitivity percentage [36]. To avoid duplicates of isolates, the first bacterial isolate for every patient throughout 365 days was considered, regardless of the sample body site or profile of antibiotic sensitivity. For example, if a patient undergoes several susceptibility tests for the same bacterial species in the same year, only the first test will be considered, irrespective of the test results and the type of specimen. After the data were received, it underwent a thorough review procedure to ensure that the right bacterial species from various facilities were combined with the appropriate susceptibility test. Afterward, the data were transferred to a clear registry, so that big data technologies could be used to perform the last analysis. After comparing the test results with the culture records kept in each microbiology department, this step led to merging the test results for a few bacterial species (Table S7). This was carried out to ensure that the aggregation would provide accurate readings despite facility differences when recording the bacterial subspecies. Finally, listing the bacterial species and antimicrobial agents followed the CLSI cumulative data presentation protocols [36].

## 5. Conclusions

The aim of presenting these data is to provide actionable recommendations for policymakers at Al-Karak Hospital to optimize antibiotic use and enhance antimicrobial stewardship. Based on the findings of the current research, the following recommendations can be given and as follows:

1. Prioritize using high-efficacy antibiotics, such as amikacin, nitrofurantoin, and piperacillin + tazobactam, as first-line options for empiric treatment of susceptible infections.
2. De-prioritize cefuroxime, ceftriaxone, and amoxicillin + clavulanic acid for severe infections due to their lower effectiveness in *E. coli* and *K. pneumoniae*.
3. Strengthen testing for reserve antibiotics: ensure routine susceptibility testing for critical antibiotics like colistin, tigecycline, and ceftazidime + avibactam to improve diagnostic accuracy and avoid false negatives.
4. Focus on resistant strains: perform genomic sequencing on extensively drug-resistant (XDR) and pan-drug-resistant (PDR) strains of *P. aeruginosa* and *K. pneumoniae* to identify resistance mechanisms and guide targeted interventions.
5. Optimize treatment for Gram-positive infections: limit the empirical use of ciprofloxacin and cefuroxime for *E. coli* and *K. pneumoniae* infections and prioritize alternatives to clindamycin for *S. viridans*, considering susceptibility data.
6. Ensure the best laboratory diagnostic practices: improve diagnostic accuracy to avoid bias in pathogen detection, sample blood-born infection, and do antimicrobial susceptibility testing.

These recommendations aim to improve treatment outcomes, reduce antimicrobial resistance, and promote sustainable antibiotic practices at the hospital.

## Figures and Tables

**Table 1:** The distribution of bacterial isolates according to sampling sources and percentages relative to the total number of isolates was reported in Al-Karak Governmental Hospital in 2022.

Sources of Bacterial Isolates	N	%
Urine (Gram-positive isolates)	69	5.81%
Urine (Gram-negative isolates)	655	55.18%
Subtotal (Urine isolates)	724	60.99%
Non-urine isolates	463	39%
Grand total	1187	100%

Non-urine includes isolates from blood, vagina, skin, and respiratory tract, among others.

**Table 2:** The antibiotic susceptibility pattern of Gram-positive bacterial isolates originated from tested urine specimens subjected to antimicrobial testing, and the percentage of susceptibility (*n*, %) was calculated to the total (N) tested isolated for the corresponding antibiotic shown. Antibiotics are shown classified according to the Access-Watch-Reserve scheme.

Antibiotic/ AWaRe Class	*E. faecalis* N	*E. faecalis* *n* (%)	*S. viridans* (α) N	*S. viridans* (α) *n* (%)	CoNS N	CoNS *n* (%)
1-Access						
Penicillin G	11	11 (100%)	-	-	-	-
Ampicillin	24	23 (96%)	11	9 (82%)	-	-
Oxacillin	-	-	17	9 (53%)	14	2(14%)
Clindamycin	R	R	25	11 (44%)	15	7 (47%)
Gentamicin	R	R	21	17 (81%)	19	18 (95%)
Tetracycline	23	2 (9%)	-	-	-	-
Nitrofurantoin	21	21 (100%)	12	8 (67%)	14	13 (93%)
Trimethoprim + Sulfamethoxazole	R	R	15	6 (40)	16	10 (63%)
2-Watch						
Ciprofloxacin	22	16 (73)	16	11 (69)	13	11 (85%)
Cefotaxime	R	R	16	13 (81%)	R	R
Levofloxacin	22	17 (77%)	22	18 (82%)	17	16 (94%)
Erythromycin	22	2 (9%)	25	10 (40%)	18	5 (28%)
Vancomycin	25	24 (96%)	S1	100%	S2	99%
Teicoplanin	-	-	12	12 (100%)	11	11 (100%)
3-Reserved						
Linezolid	S2	99%	S1	100%	S2	99%
Statistics of reported numbers (N)						
Minimum (N)	11		11		11	
Average (N)	18		18		15	
Maximum (N)	25		25		19	
Total (Gram-positive, Urine)		69				

S1: Expected to be 100% intrinsically susceptible; S2: Expected to be 99% intrinsically susceptible; R: Expected resistance; (-): not being tested sufficiently or isolates were <10.

**Table 3:** The antibiotic susceptibility pattern of Gram-negative bacterial isolates originated from tested urine specimens subjected to antimicrobial testing and the percentage of susceptibility (*n*, %) calculated to the total (N) tested isolates for the corresponding antibiotic shown. Antibiotics are shown classified according to the Access-Watch-Reserve scheme.

Antibiotic/AWaRe Class	*E. coli* N	*E. coli* *n* (%)	*K. pneumoniae* N	*K. pneumoniae* *n* (%)
1-Access				
Amoxicillin + Clavulanic	255	140 (55%)	19	12 (63%)
Cefazolin	161	86 (42%)	12	5 (42%)
Amikacin	501	466 (93%)	36	29 (81%)
Gentamicin	615	547 (89%)	40	35 (88%)
Nitrofurantoin	386	332 (86%)	23	7 (30%)
Trimethoprim + Sulfamethoxazole	563	265 (47%)	36	19 (53%)
2-Watch				
Ceftazidime	594	422 (71%)	40	22 (55%)
Cefuroxime	451	194 (43%)	26	8 (31%)
Ceftriaxone	437	240 (55%)	27	13 (48%)
Cefepime	339	251 (74%)	23	11 (48%)
Piperacillin + Tazobactam	426	392 (92%)	26	21 (81%)
Ciprofloxacin	463	218 (47%)	31	15 (48%)
Levofloxacin	115	79 (69%)	-	-
Imipenem	S2	99%	S2	99%
Meropenem	S2	99%	S2	99%
Ertapenem	S2	99%	S2	99%
3-Reserved				
Ceftolozane + Tazobactam	62	58 (94%)	-	-
Ceftazidime + Avibactam	S2	99%	S2	99%
Colistin	S1	100%	S1	100%
Fosfomycin	226	215 (95%)	15	12 (80%)
Tigecycline	12	12 (100%)	-	-
Statistics of reported numbers (N)				
Minimum (N)	12		12	
Average (N)	313.5		26	
Maximum (N)	615		40	
Total (Gram-negative, Urine)		655		

S1: Expected to be 100% intrinsically susceptible; S2: Expected to be 99% intrinsically susceptible; R: Expected resistance; (-): not being tested sufficiently or isolates were <10.

**Table 4:** The table presents a summary of possible extensive drug-resistant (XDR) and pan-drug-resistant (PDR) among all isolates reported in the “Antibiogram, Al-Karak Hospital, Ministry of Health, 2022” report.

*Pathogen*	Isolates from All Specimens N	XDR n (%)	PDR n (%)
*P. aeruginosa*	53	8 (15.1%)	3 (5.7%)
*K. pneumoniae*	62	7 (11.3%)	2 (3.2%)
*E. coli*	722	28 (3.8%)	4 (0.6%)
Subtotal (all specimens Gram-negative)	837	43 (5.1%)	9 (1.1%)
*E. faecalis*	38	0	0
CoNS	52	2 (3.9%)	0
*S. viridans* (α)	69	2 (2.9%)	0
*S. aureus*	76	1 (1.3%)	1 (1.3%)
Subtotal (all specimens Gram-positive)	235	5 (2.1%)	1 (0.4%)
Other pathogens	153	N/A	N/A
Grand total	1187	N/A	N/A

N/A: Data are unavailable in the original “Antibiogram, Al-Karak Hospital, Ministry of Health, 2022” report.

**Table 5:** Comparison of selected cases from the current study to previous studies regarding the susceptibility of Gram-positive isolates, a full version of this table is found in the Supplementary Information.

Antibiotic	Current Study		Previous Study		Susceptibility of Current vs. Previous Study	Reference
	N	*n* (%)	N	*n* (%)		
*E. faecalis*						
Erythromycin	22	2 (9%)	159	5 (62.5%)	Lower	[11]
Vancomycin	25	24 (96%)	159	8 (100%)	Lower	[11]
Ciprofloxacin	22	16 (73%)	19	7 (57.9%)	Higher	[12]
Levofloxacin	22	17 (77%)	19	8 (57.9%)	Higher	[12]
*S. viridans* (α)						
Erythromycin	25	10 (40%)	28	11 (61%)	Lower	[13]
Levofloxacin	22	18 (82%)	28	10 (64.3%)	Higher	[13]
Clindamycin	25	11 (44%)	28	3 (89%)	Lower	[13]
CoNS						
Oxacillin	14	2 (14%)	233	(24.2%)	Lower	[14]
Erythromycin	18	5 (28%)	30	(8.3%)	Higher	[15]

**Table 6:** Comparison of selected cases from the current study to previous studies regarding the susceptibility of Gram-negative isolates, a full version of this table is found in the Supplementary Information.

Antibiotic	Current Study		Previous Study		Susceptibility of Current vs. Previous Study	Reference
	N	*n* (%)	N	*n* (%)		
*E. coli*						
Cefuroxime	451	194 (43%)	177	107 (60.5%)	Lower	[16]
Ciprofloxacin	463	218 (47%)	154	116 (75.8%)	Lower	[16]
Nitrofurantoin	386	332 (86%)	178	133 (75%)	Higher	[16]
Tigecycline	12	12 (100%)	5	3 (60%)	Higher	[16]
Piperacillin + Tazobactam	426	392 (92%)	68	65 (95.7%)	Lower	[16]
*K. pneumoniae*						
Piperacillin + Tazobactam	26	21 (81%)	24	24 (100%)	Lower	[16]
Cefuroxime	26	8 (31%)	42	21 (50%)	Lower	[16]
Ciprofloxacin	31	15 (48%)	37	36 (97.3%)	Lower	[16]
Nitrofurantoin	23	7 (30%)	28	9 (20.9%)	Higher	[16]

## Data Availability

The study dataset used in this study is available from the corresponding author upon reasonable request.

## Supplementary Materials

The following supporting information can be downloaded at: https://www.mdpi.com/article/10.3390/antibiotics13121172/s1. Table S1: Susceptibility pattern coagulase-negative *Staphylococci* isolated from blood specimens at Al-Karak Hospital, 2022; Table S2: Comparison of antibiotic susceptibility percentages for *E. faecalis* between current and previous studies; Table S3: Comparison of antibiotic susceptibility percentages for *S. viridans* (α) between current and previous studies; Table S4: Comparison of antibiotic susceptibility percentages for CoNS (Coagulase Negative *Staphylococci*) between current and previous studies; Table S5: Comparison of antibiotic susceptibility percentages for *E. coli* between current and previous studies; Table S6: Comparison of antibiotic susceptibility percentages for *K. pneumoniae* between current and previous studies; Table S7: The convention used in the current study to report pathogens. Refs. [37,38,39,40,41,42,43] are cited in Supplementary Materials.
