# Supplementary material for: Monitoring of Antibiotic Resistance Patterns Within Al-Karak Governmental Hospital, Jordan, in 2022
Source: Antibiotics (Basel). 2024 Dec 4;13(12):1172. doi: 10.3390/antibiotics13121172 (PMC11672563; doi:10.3390/antibiotics13121172)
Supplement: Supplementary file 1 [file antibiotics-13-01172-s001.zip › antibiotics-3296523-supplementary.pdf]

Supplementary Information

Monitoring of Antibiotic Resistance Patterns within Al Karak Governmental Hospital, Jordan, in 2022

Amin A. Aqel [1], Tala M. Al-matarneh [2], Tayf K. Al-tarawneh [3], Tahrir Al-nawayseh [4], Mohammed Alsbou [5,6,*], Yasser Gaber [3,7]

1 Department of Microbiology and Immunology, Faculty of Medicine, Mutah University, Jordan; aminaq@mutah.edu.jo;
2 Pharmacology and Toxicology Department Faculty of Pharmacy, Cairo University, Cairo, Egypt.; Ta-la.Matarneh@yahoo.com;
3 Department of Pharmaceutics and Pharmaceutical Technology, Faculty of Pharmacy, Mutah University, Al-Karak, 61710, Jordan; taifkhaledtara@yahoo.com;
4 Head of infection prevention and control Al Karak governmental hospital, Jordan, Tahreer761@yahoo.com;
5 Department of Pathological Sciences, College of Medicine, Ajman University, United Arab Emirates. m.alsbou@ajman.ac.ae
6 Department of Pharmacology, Faculty of Medicine, Mutah University, Jordan;
7 Department of Microbiology and Immunology, Faculty of Pharmacy, Beni-Suef University, 62511, Beni-Suef, Egypt, Yasser.Gaber@pharm.bsu.edu.eg.
* Correspondence: Professor Mohammed Alsbou, m.alsbou@ajman.ac.ae

Table S1. Susceptibility pattern coagulase-negative staphylococci isolated from blood specimens at Al-Karak Hospital, 2022

**Antibiotic / AWaRe class**	**Coagulase negative *Staphylococci***	
	N	n %
**1-Access**		
Amoxicillin + Clavulanic acid	-	-
Penicillin G	-	-
Ampicillin	-	-
Clindamycin	10	6(60)
Cefazolin	-	-
Flucloxacillin	-	-
Gentamicin	17	15(88)
Nitrofurantoin	-	-
Oxacillin	-	-
Rifampin	-	-
Trimethoprim + Sulfamethoxazole	12	5(42)
Tetracycline	-	-
**2-Watch**		
Cefotaxime	-	-
Ciprofloxacin	-	-
Erythromycin	12	6(50)
Cefepime	-	-
Levofloxacin	-	-
Teicoplanin	-	-
Vancomycin	S2	99%
**3-Reserved**		
Linezolid	S2	99%
Tigecycline	-	-

S1: Expected to be 100 % intrinsically susceptible; S2: Expected to be 99 % intrinsically susceptible; R: Expected resistance; (-): not being tested sufficiently or isolates were < 10.

Table S2. Comparison of antibiotic susceptibility percentages for *E. faecalis* between current and previous studies

Antibiotic	Current study		Previous study		Susceptibility of current vs previous study	Reference
	N	n(%)	N	n(%)		
E. faecalis						
**Ampicillin**	24	23(96%)	159	8(100%)	Lower	[1]
**Erythromycin**	22	2(9%)	159	5(62.5%)	Lower	[1]
**Vancomycin**	25	24(96%)	159	8(100%)	Lower	[1]
**Ciprofloxacin**	22	16(73%)	19	7(57.9%)	Higher	[2]
**Levofloxacin**	22	17(77%)	19	8(57.9%)	Higher	[2]

Table S3. Comparison of antibiotic susceptibility percentages for *S. viridans* (α) between current and previous studies

Antibiotic	Current study		Previous study		Susceptibility of current vs previous study	Reference
	N	n(%)	N	n(%)		
***S. viridans* (α)**						
**Ampicillin**	11	9(82%)	81	9(89%)	Lower	[3]
**Erythromycin**	25	10(40%)	28	11(61%)	Lower	[4]
**Vancomycin**	S1	100%	28	2(92.9%)	Higher	[4]
**Levofloxacin**	22	18(82%)	28	10(64.3%)	Higher	[4]
**Clindamycin**	25	11(44%)	28	3(89%)	Lower	[4]

Table S4. Comparison of antibiotic susceptibility percentages for CoNS (Coagulase Negative Staphylococci) between current and previous studies

Antibiotic	Current study		Previous study		Susceptibility of current vs previous study	Reference
	N	n(%)	N	n(%)		
**CoNS**						
**Oxacillin**	14	2(14%)	233	(24.2%)	Lower	[5]
**Erythromycin**	18	5(28%)	30	(8.3%)	Higher	[6]
**Vancomycin**	S2	99%	223	(100%)	Almost similar	[5]
**Ciprofloxacin**	13	11(85%)	32	28(80%)	Higher	[7]
**Levofloxacin**	17	16(94%)	30	(91.7)	Higher	[6]
**Clindamycin**	15	7(47%)	30	(50%)	Lower	[6]
**Nitrofurantoin**	14	13(93%)	131	115(87.9%)	Higher	[7]
**Trimethoprim + Sulfamethoxazole**	16	10(63%)	129	88(68.2%)	Lower	[7]
**Gentamicin**	19	18(95%)	126	117(91.4%)	Higher	[7]
**Linezolid**	S2	99%	223	(100%)	Almost similar	[5]

Table S5. Comparison of antibiotic susceptibility percentages for *E. coli* between current and previous studies

Antibiotic	Current study		Previous study		Susceptibility of current vs previous study	Reference
	N	n(%)	N	n(%)		
E. coli						
**Carbapenems**	S2	99%	132	99%	Almost similar	[8]
**Carbapenems**	S2	99%	ESBL 251	(96-99%)	Almost similar	[9]
**Carbapenems**	S2	99%	64	(95-100%)	Almost similar	[10]
**Carbapenems**	S2	99%	150	(90-100%)	Almost similar	[11]
**Cefuroxime**	451	194(43%)	ESBL 63 Non-ESBL 69	ESBL-1% Non-ESBL 60%	Footnote[1]	[8]
**Cefuroxime**	451	194(43%)	177	107(60.5%)	Lower	[7]
**Cefepime**	339	251(74%)	150	75(50%)	Higher	[11]
**Cefepime**	339	251(74%)	83	53(63.8%)	Higher	[7]
**Ceftriaxone**	437	240(55%)	85	45(52.9%)	Higher	[7]
**Ceftazidime**	594	422(71%)	163	95(62.1%)	Higher	[7]
**Amikacin**	501	466(93%)	84	71(84.5%)	Higher	[7]
**Gentamicin**	615	547(89%)	180	152(84.4%)	Higher	[7]
**Ciprofloxacin**	463	218(47%)	154	116(75.8%)	Lower	[7]
**Nitrofurantoin**	386	332(86%)	178	133(75%)	Higher	[7]
**Trimethoprim + Sulfamethoxazole**	563	265(47%)	180	101(55.1%)	Lower	[7]
**Tigecycline**	12	12(100%)	5	3(60%)	Higher	[7]
**Piperacillin + Tazobactam**	426	392(92%)	68	65(95.7%)	Lower	[7]

[1]Higher compared to ESBL-producing strains but lower susceptibility compared to non-ESBL-producing strains.

Table S6. Comparison of antibiotic susceptibility percentages for *K. pneumoniae* between current and previous studies

Antibiotic	Current study		Previous study		Susceptibility of current vs previous study	Reference
	N	n(%)	N	n(%)		
K. pneumoniae						
**Carbapenems**	S2	99%	ESBL 251	(96-99%)	Almost similar	[9]
**Carbapenems**	S2	99%	296	(98%)	Almost similar	[12]
**Carbapenems**	S2	99%	11	(95%-100%)	Almost similar	[10]
**Carbapenems**	S2	99%	15	(90%)	Higher	[13]
**Piperacillin + Tazobactam**	26	21(81%)	24	24(100%)	Lower	[7]
**Cefuroxime**	26	8(31%)	42	21(50%)	Lower	[7]
**Ceftriaxone**	27	13(48%)	15	8(53.3%)	Lower	[7]
**Ceftazidime**	40	22(55%)	37	18(48.6%)	Higher	[7]
**Cefepime**	23	11(48%)	14	7(50%)	Lower	[7]
**Amikacin**	36	29(81%)	14	14(100%)	Lower	[7]
**Gentamicin**	40	35(88%)	43	35(81.4%)	Higher	[7]
**Ciprofloxacin**	31	15(48%)	37	36(97.3%)	Lower	[7]
**Nitrofurantoin**	23	7(30%)	28	9(20.9%)	Higher	[7]
**Trimethoprim + Sulfamethoxazole**	36	19(53%)	56	35(58.1%)	Lower	[7]

Table S7. The convention used in the current study to report pathogens

Recorded Bacterial species	Reported Bacterial species
Klebsiella spp. or Klebsiella ESBL or *Klebsiella pneumoniae*	*K. pneumoniae*
Escherichia coli (O157); or *Escherichia coli ESBL*; or *Escherichia coli*	*E. coli*
Enterobacter spp. or *Enterobacter ESBL* or *Enterobacter cloacae*	*E. cloacae*
*Acinetobacte*r *spp*.; or *Acinetobacter baumannii*	*A. baumanni*
Citrobacter spp.; Citrobacter freundii	*C. freundii*
Staphylococcus capitis; Staphylococcus epidermidis; Staphylococcus homing or *Staphylococcus saprophyticus*	CoNS
Streptococcus (β-Hemolytic) Group A or Streptococcus pyogenes	*S. pyogenes*
Streptococcus (α-Hemolytic) or Streptococcus viridans	*S. viridans*
Pseudomonas spp.	*P. aeruginosa*
Enterococcus faecalis; or Enterococcus faecalis (Group D)	*E. faecalis*

References

1. Salah, R.; Dar-Odeh, N.; Abu Hammad, O.; Shehabi, A.A. Prevalence of putative virulence factors and antimicrobial susceptibility of Enterococcus faecalis isolates from patients with dental Diseases. *BMC Oral Health* **2008**, *8*, 17, doi:10.1186/1472-6831-8-17.
2. Luty, R.S.; Fadil, A.G.; Najm, J.M.; Abduljabbar, H.H.; Kashmar, S.A.A. Uropathogens antibiotic susceptibility as an indicator for the empirical therapy used for urinary tract infections: a retrospective observational study. *Iran J Microbiol* **2020**, *12*, 395-403, doi:10.18502/ijm.v12i5.4599.
3. Abu-Zineh, R.; Dar-Odeh, N.; Shehabi, A. Macrolide resistance genes and virulence factors of common viridans Streptococci species colonizing oral cavities of patients in Jordan. *Oral Health Dent Manag* **2015**, *14*, 337-341.
4. ALZOUBI, H.; ABU-LUBAD, M.; AL-MNAYYIS, A.A.; SATARI, A.; ALZOBI, M.; AL RAMADNEH, M.; JARAJREH, D.A. Effect of Electronic Cigarettes on the Carriage of Selected Organisms in the Nasal and Oral Cavity in Comparison to Tobacco Smokers and Non-smokers. *Journal of Clinical & Diagnostic Research* **2020**, *14*.
5. Al Tayyar, I.A.; Al-Zoubi, M.S.; Hussein, E.; Khudairat, S.; Sarosiekf, K. Prevalence and antimicrobial susceptibility pattern of coagulase-negative staphylococci (CoNS) isolated from clinical specimens in Northern of Jordan. *Iran J Microbiol* **2015**, *7*, 294-301.
6. Al-Tamimi, M.; Abu-Raideh, J.; Himsawi, N.; Khasawneh, A.; Hawamdeh, H. Methicillin and vancomycin resistance in coagulase-negative Staphylococci isolated from the nostrils of hospitalized patients. *The Journal of Infection in Developing Countries* **2020**, *14*, 28-35, doi:10.3855/jidc.11025.
7. Matalka, A.; Al-Husban, N.; Alkuran, O.; Almuhaisen, L.; Basha, A.; Eid, M.; Elmuhtaseb, M.S.; Al Oweidat, K. Spectrum of uropathogens and their susceptibility to antimicrobials in pregnant women: a retrospective analysis of 5-year hospital data. *J Int Med Res* **2021**, *49*, 3000605211006540, doi:10.1177/03000605211006540.
8. Albaramki, J.H.; Abdelghani, T.; Dalaeen, A.; Khdair Ahmad, F.; Alassaf, A.; Odeh, R.; Akl, K. Urinary tract infection caused by extended-spectrum β-lactamase-producing bacteria: Risk factors and antibiotic resistance. *Pediatr Int* **2019**, *61*, 1127-1132, doi:10.1111/ped.13911.
9. Almomani, B.A.; Hayajneh, W.A.; Ayoub, A.M.; Ababneh, M.A.; Al Momani, M.A. Clinical patterns, epidemiology and risk factors of community-acquired urinary tract infection caused by extended-spectrum beta-lactamase producers: a prospective hospital case-control study. *Infection* **2018**, *46*, 495-501, doi:10.1007/s15010-018-1148-y.
10. Hirmas, N.; Mubarak, S.; Sultan, I. Patterns of microbial growth in urine cultures in a pediatric hematology/oncology unit over a one-year period: a single institution study. *International Journal of Pediatrics and Adolescent Medicine* **2017**, *4*, 95-99.
11. Al-Tamimi, M.; Abu-Raideh, J.; Albalawi, H.; Shalabi, M.; Saleh, S. Effective oral combination treatment for extended-spectrum beta-lactamase-producing Escherichia coli. *Microbial Drug Resistance* **2019**, *25*, 1132-1141.

12. Aqel, A.A.; Giakkoupi, P.; Alzoubi, H.; Masalha, I.; Ellington, M.J.; Vatopoulos, A. Detection of OXA-48-like and NDM carbapenemases producing Klebsiella pneumoniae in Jordan: A pilot study. *Journal of Infection and Public Health* **2017**, *10*, 150-155, doi:10.1016/j.jiph.2016.02.002.
13. Yusef, D.; Jahmani, T.; Kailani, S.; Al-Rawi, R.; Khasawneh, W.; Almomani, M. Community-acquired serious bacterial infections in the first 90 days of life: a revisit in the era of multi-drug-resistant organisms. *World Journal of Pediatrics* **2019**, *15*, 580-585, doi:10.1007/s12519-019-00276-w.
